# A Review of Remarks on the Professional Education of Dentists

**Published:** 1852-10

**Authors:** 


*A Review of Remarks on the Professional Education of Dentists;* by John Trenor, M. D., Dentist of New York. By Amos Westcott, M. D., Professor of Theory and Practice in the New York College of Dental Surgery, at Syracuse.

Our readers are well informed of our opinion of the system advocated by Dr. Trenor, or rather, the fantastical experiment he suggests. Dr. Westcott, who like ourselves, commenced his preparation for dental practice in a medical school, and consequently found himself a doctor before he had made the first approaches towards the qualifications of a dentist, naturally feels indignant at Dr. Trenor's programme of a fancy dental school. He goes into the controversy with great advantages over his antagonist. He has experience, good sense and a scientific knowledge of dentistry, and being determined to give Dr. Trenor a satisfactory chastisement, he does his work with an unction quite edifying.

We begin to pity Dr. Trenor. Perhaps after all, his scheme was not intended to be seriously put in practice. Perhaps his supplementary medical professorships were not intended to be real, but something like what the Scotch call *Tulchans*—calf skins stuffed with straw placed under a cow to coax her to let down her milk.

